# Supplementary material for: Global trends and disparities in ischemic heart disease attributable to high systolic blood pressure, 1990–2021: Insights from the global burden of disease study
Source: PLoS One. 2025 Jun 12;20(6):e0324073. doi: 10.1371/journal.pone.0324073 (PMC12161563; doi:10.1371/journal.pone.0324073)
Supplement: Attachment — Submitted filename: Response to Reviewers.pdf [file pone.0324073.s005.pdf]

Response to Reviewers

Journal Requirements:

When submitting your revision, we need you to address these additional requirements.

1. Please ensure that your manuscript meets PLOS ONE's style requirements, including those for file naming. The PLOS ONE style templates can be found at https://journals.plos.org/plosone/s/file?id=wjVg/PLOSOne_formatting_sample_main_body.pdf and https://journals.plos.org/plosone/s/file?id=ba62/PLOSOne_formatting_sample_title_authors_affiliations.pdf

Re: The authors have standardized the formatting of the manuscript according to the template provided by the journal.

2. We note that Figure 4 in your submission contain [map/satellite] images which may be copyrighted. All PLOS content is published under the Creative Commons Attribution License (CC BY 4.0), which means that the manuscript, images, and Supporting Information files will be freely available online, and any third party is permitted to access, download, copy, distribute, and use these materials in any way, even commercially, with proper attribution. For these reasons, we cannot publish previously copyrighted maps or satellite images created using proprietary data, such as Google software (Google Maps, Street View, and Earth). For more information, see our copyright guidelines: http://journals.plos.org/plosone/s/licenses-and-copyright.

We require you to either (1) present written permission from the copyright holder to publish these figures specifically under the CC BY 4.0 license, or (2) remove the figures from your submission.

Re: We have sought permission from the original copyright owner of Figure 4 to distribute the content under a CC BY 4.0 license. We contacted the original copyright owner with a content permission form (http://journals.plos.org/plosone/s/file?id=7c09/content-permission-form.pdf) and the following text:

“I am seeking permission to use the map resources from the website https://www.resdc.cn/data.aspx?DATAID=205. I intend to use the map I intend to use the map image resources for creating figures in my papers. I request permission to publish the following map images in the open - access journal PLOS ONE under the Creative Commons Attribution License. Creative Commons Attribution License (CCAL) CC BY 4.0. Please be aware that this license allows unrestricted use and distribution, even commercially, by third parties.”

I am uploading the completed Content License Form as an “Other” file. In the caption of Figure 4, we wrote: “This map was created with permission from Xin-Liang Xu, using map resources at ‘https://www.resdc.cn/data.aspx?DATAID=205’, original copyright 2025.”

Additional Editor Comments:

This is a epidemiologic study using GBD database. The topic is interesting as global disparity of management and burden of hypertension-related IHD is an important challenge for public health. However, methodology and discussion section might need some major revisions. My comments are included.

Methodology section:

1. The authors should include more details about their approach: How did you extract the data? Which GBD estimate did you use? Which age groups did you include? Did you restrict to adult or include all age?

Re: Thank you for your insightful comments. We have added more details about our approach in the Data Collection section (lines 155-164 & 170-173).

2. How did you determine the mortality of hypertension-related IHD? It would be very helpful to indicate the method precisely for readers who are not familiar with GBD can understand.

Re: Thank you for your insightful comments. We supplemented the Data Source section with methods for estimating mortality and DALYs for HSBP-related IHD (lines 108-132).

3. A brief summary of statistic model using by GBD Collaborators to establish those datasets/estimate would also be helpful.

Re: Thank you for your insightful comments. In the Materials and Methods section, we

briefly describe the statistical models used by the GBD collaborators to establish these datasets/estimates, including the Cause of Death Ensemble model (CODem), DisMod-MR 2.1 (a Bayesian disease modeling meta-regression tool), population attributable fraction (PAF), and ASR.

4. Statistical analysis: How did you calculate EAPC? Which model did you use? A details explanation would be needed.

Re: Thank you for your insightful comments. We provide a detailed description of the statistical modeling of EAPC in the Statistical Analysis section (lines 178-181).

Discussion section:

This section is overall confusing with repetitive statement.

1. From line 281 - 293. The statement that hypertension-related IHD is dominant in male than female was mentioned in line 281 then repeated in 286-287. Moreover, lines 283-285 and lines 287-290 had literally the same meaning.

Re: Thank you for your insightful comment. Based on the suggestions from both the editor and reviewers, we conducted a careful review of the discussion section and refined lines 281–293. Redundant statements regarding sex differences and hormonal mechanisms were consolidated to improve clarity and ensure the discussion remains focused and non-repetitive (lines 353-366).

2. You need to discuss more about the underlying reasons of your findings (disparity

in sex/age/region) and propose potential solution.

Re: Thank you for your insightful suggestion. In response to your suggestion and in alignment with feedback from other reviewers, we have expanded the discussion to explore the potential biological, behavioral, and systemic drivers behind the observed disparities by sex, age, and region. We also propose tailored public health strategies, including earlier screening in high-risk groups, community-based interventions in low-resource settings, and age-specific prevention efforts for younger populations (lines 345-455).

3. Limitation (lines 307-317). You need to acknowledge that hypertension defined by GBD is significantly different to the current definition from major guidelines including AHA/ACC, ESC, JNC, ISH.... This definition did overestimated your findings concerning the burden of IHD.

Re: We appreciate your valuable feedback. We acknowledge that the GBD definition of HSBP differs significantly from major clinical guidelines, including AHA/ACC (≥130/80 mmHg) [1], ESC, JNC, and ISH (≥140/90 mmHg) [2-4]. Unlike these guideline-based definitions, GBD models HSBP as a continuous risk factor rather than a categorical diagnosis, and in GBD 2021, the theoretical minimum risk exposure level (TMREL) was revised from 110–115 mmHg to 105–115 mmHg.

To address this concern and avoid potential misinterpretation, we have clarified in the Introduction that the definition of HSBP used by the GBD differs from standard clinical

diagnostic thresholds in major guidelines. Additionally, we explicitly acknowledge this distinction in the Limitations section, noting that the GBD modeling approach may overestimate the attributable burden compared to guideline-defined hypertension.

References

[1]. Whelton PK, Carey RM, Aronow WS, et al. 2017 ACC/AHA/AAPA/ABC/ACPM/AGS/APhA/ASH/ASPC/NMA/PCNA Guideline for the Prevention, Detection, Evaluation, and Management of High Blood Pressure in Adults: A Report of the American College of Cardiology/American Heart Association Task Force on Clinical Practice Guidelines. Circulation. 2018;138(17):e484-e594.

[2]. James PA, Oparil S, Carter BL, et al. 2014 evidence-based guideline for the management of high blood pressure in adults: report from the panel members appointed to the Eighth Joint National Committee (JNC 8) [published correction appears in JAMA. 2014 May 7;311(17):1809]. JAMA. 2014;311(5):507-520.

[3]. Unger T, Borghi C, Charchar F, et al. 2020 International Society of Hypertension global hypertension practice guidelines. J Hypertens. 2020;38(6):982-1004.

[4]. McEvoy JW, McCarthy CP, Bruno RM, et al. 2024 ESC Guidelines for the management of elevated blood pressure and hypertension [published correction appears in Eur Heart J. 2025 Feb 11:ehaf031.

Reviewers' comments:

Reviewer #1:

Deferring line edits for now as an epidemiologic study of ischemic heart disease without statistical analyses and/or adjustments including smoking, lipids, or diabetes does not, to me, seem technically sound or scientifically rigorous.

Re: We sincerely appreciate your valuable comments on our study. In response to your concerns, we would like to clarify our research methodology and provide further evidence of the scientific rigor and validity of our study.

1) This Study is Based on GBD 2021 Data and Employs Rigorous Statistical Analyses

Our study utilizes data from the Global Burden of Disease Study 2021 (GBD 2021), one of the most comprehensive and methodologically robust epidemiological studies worldwide. The GBD 2021 dataset is derived from over 100,000 data sources, incorporating population registries, health surveys, and clinical data, and is analyzed using advanced statistical modeling techniques.

The GBD 2021 analytical framework includes: (1) Data collection and integration: Combining multiple data sources, including health records and surveys. (2) Statistical modeling and estimation: Applying hierarchical regression models and Bayesian meta-regression (DisMod-MR 2.1) to adjust for uncertainty [1]. (3) Attributable risk analysis: Employing Comparative Risk Assessment (CRA) methodology to evaluate the impact of different risk factors and adjust for confounding variables [2]. Thus, contrary to the concern that the study lacks statistical analysis, our findings are based on scientifically

robust, globally recognized methodologies.

2) Our Study Fully Accounts for and Adjusts Key Risk Factors, Including Smoking, Lipids, and Diabetes

GBD 2021 explicitly considers smoking, high low-density lipoprotein cholesterol (LDL-C), and diabetes as major risk factors and adjusts for them in its data analysis. Specifically, GBD 2021 applies attributable risk estimation methods, integrating exposure level assessments and relative risk calculations to quantify each risk factor's contribution to ischemic heart disease (IHD) [2]. Previous GBD studies have extensively analyzed smoking, high LDL-C, and high fasting glucose (FPG), which are considered major risk factors for IHD with well-documented trends in global burden [3-5]. Thus, our study's statistical methodology has already incorporated and adjusted for these major risk factors, ensuring a rigorous and accurate analysis.

3) Previous Studies Have Reported Global Attributable Burden of These Risk Factors but Only at an Aggregate Level

Numerous prior studies have assessed the global burden of smoking, high LDL-C, and high FPG on IHD [6,7]. For example, a comprehensive analysis of IHD burden across 204 countries from 1990 to 2019 identified hypertension, high LDL-C, and smoking as primary risk factors, with an extensive global report [6]. However, these studies primarily focus on global and broad regional trends and lack detailed stratification by specific populations, such as different age groups, sexes, and geographic regions [7].

Our Study's Novel Contribution: (1) Refines the analysis of attributable risk factors: Moving beyond global-level reporting, our study delves deeper into risk patterns across

diverse demographic and geographic segments. (2) Enhances methodological rigor: Ensuring that findings are not only globally relevant but also actionable for targeted public health interventions. Therefore, our study is not lacking in rigor but rather expands upon existing research by providing more granular insights.

4) Extensive Literature Supports the Scientific Validity of Our Study Design

Numerous epidemiological studies have adopted GBD methodologies to examine single-risk factor contributions to IHD, and these studies have been widely recognized in leading journals [3-5]. Studies have individually analyzed the impact of high LDL-C, smoking, and high FPG on IHD burden, employing similar statistical modeling approaches [3-5]. Additionally, attributable burden studies of high systolic blood pressure (HSBP) on cardiovascular diseases, such as atrial fibrillation and stroke, have been extensively conducted, further validating our analytical framework [8,9]. The GBD 2021 research methodology is endorsed by the World Health Organization (WHO), United Nations (UN), and World Bank, establishing it as the gold standard for global disease burden estimation [1].

Our Study Aligns with Established Scientific Standards: (1) Strict adherence to the Guidelines for Accurate and Transparent Health Estimates Reporting (GATHER) to ensure transparency and reproducibility. (2) Utilization of standardized GBD analytical frameworks, incorporating cutting-edge statistical models and risk factor assessments. Thus, our study design is fully aligned with established scientific methodologies and carries significant public health relevance.

In summary, our study:

·Is based on GBD 2021 data and employs internationally recognized statistical methodologies.

·Fully accounts for and adjusts key risk factors, including smoking, high LDL-C, and diabetes.

·Advances beyond prior studies by providing a more detailed analysis of risk burden at regional and demographic levels.

·Follows an analytical framework validated by extensive prior literature and leading global health organizations.

We believe this clarification addresses your concerns, and we sincerely appreciate your constructive feedback. We look forward to your further comments.

References

[1] GBD 2021 Diseases and Injuries Collaborators. Global incidence, prevalence, years lived with disability (YLDs), disability-adjusted life-years (DALYs), and healthy life expectancy (HALE) for 371 diseases and injuries in 204 countries and territories and 811 subnational locations, 1990-2021: a systematic analysis for the Global Burden of Disease Study 2021. Lancet. 2024;403(10440):2133-2161. doi:10.1016/S0140-6736(24)00757-8.

[2] GBD 2021 Risk Factors Collaborators. Global burden and strength of evidence for 88 risk factors in 204 countries and 811 subnational locations, 1990-2021: a systematic analysis for the Global Burden of Disease Study 2021. Lancet. 2024;403(10440):2162-2203. doi:10.1016/S0140-6736(24)00933-4.

[3] Zhang L, Tong Z, Han R, et al. Global, Regional, and National Burdens of Ischemic

Heart Disease Attributable to Smoking From 1990 to 2019. J Am Heart Assoc. 2023;12(3):e028193. doi:10.1161/JAHA.122.028193.

[4] Du H, Shi Q, Song P, et al. Global Burden Attributable to High Low-Density Lipoprotein-Cholesterol From 1990 to 2019. Front Cardiovasc Med. 2022;9:903126. Published 2022 Jun 9. doi:10.3389/fcvm.2022.903126.

[5] Shen N, Liu J, Wang Y, et al. The global burden of ischemic heart disease attributed to high fasting plasma glucose: Data from 1990 to 2019. Heliyon. 2024;10(5):e27065. Published 2024 Mar 6. doi:10.1016/j.heliyon.2024.e27065.

[6] Safiri S, Karamzad N, Singh K, et al. Burden of ischemic heart disease and its attributable risk factors in 204 countries and territories, 1990-2019. Eur J Prev Cardiol. 2022;29(2):420-431. doi:10.1093/eurjpc/zwab213.

[7] Wang Y, Li Q, Bi L, Wang B, Lv T, Zhang P. Global trends in the burden of ischemic heart disease based on the global burden of disease study 2021: the role of metabolic risk factors. *BMC Public Health*. 2025;25(1):310. Published 2025 Jan 24. doi:10.1186/s12889-025-21588-9.

[8] Jin Y, Wang K, Xiao B, et al. Global burden of atrial fibrillation/flutter due to high systolic blood pressure from 1990 to 2019: estimates from the global burden of disease study 2019. *J Clin Hypertens (Greenwich)*. 2022;24(11):1461-1472. doi:10.1111/jch.14584.

[9] Li J, Zhong Q, Yuan S, Zhu F. Global burden of stroke attributable to high systolic blood pressure in 204 countries and territories, 1990-2019. Front Cardiovasc Med. 2024;11:1339910. Published 2024 Apr 26. doi:10.3389/fcvm.2024.1339910.

Some more general methodologic/analytic thoughts as below:

--I see consistent with prior published work (ref. 20) and GBD TMREL, but a high SBP cut-off of 110-115 mmHg is not consistent with most clinical guidelines (many of which have changed meaningfully throughout the long study time period of 1990-2021); suggest at least mentioning in the discussion. Also, was the cut-off greater than or equal to 115 mmHg or 110 mmHg?

Re: Thank you for your thoughtful comment. We acknowledge that the definition of HSBP used in GBD differs from clinical guideline thresholds. Specifically, GBD estimates the burden of IHD attributable to SBP ≥115 mmHg, based on the comparative risk assessment framework, rather than diagnostic cut-offs used in guidelines (e.g., ≥130/80 mmHg or ≥140/90 mmHg). The theoretical minimum risk exposure level was revised in GBD 2021 to 105–115 mmHg, reflecting the SBP range associated with the lowest population-level risk. This continuous risk modeling approach facilitates burden estimation but may overestimate risk relative to clinical definitions. We have clarified this distinction in both the **Definitions** (lines 134-145) and **Limitations** (lines 425-434) sections to improve transparency.

--are SDI categories per country or per participant? It seems per country (lines 225-227 and PMID 33069327) but this should be clarified in the methods section and perhaps mentioned as a limitation in the discussion section (i.e., if SDI categories are per country, all participants from a low SDI country may not be low SDI; if SDI

categories are per participant, high SDI in one country may not align with high SDI for another country). What are the SDI categories based on?

Re: Thank you for your insightful comment regarding the classification of the Socio-Demographic Index (SDI) in our study. SDI is calculated using three key components: educational attainment (average years of schooling), income (log-transformed per capita income), and fertility rate (total fertility rate among women under 25 years old) As SDI is a widely used global metric for demographic and health studies, we followed standard methodology by categorizing participants based on the SDI level of their country rather than at an individual level.

We acknowledge that our description in the Methods and Discussion section may not have been sufficiently clear, and we have made the following revisions:

1. Clarification in the Methods Section

We have explicitly stated that SDI categories are assigned at the country level, not at the individual level. The SDI, as defined in the Global Burden of Disease (GBD) framework, is a composite measure based on income per capita, average years of education, and total fertility rate among women under 25 years old (PMID 33069327). Therefore, in our study, all participants from a given country are classified according to that country's SDI level, rather than based on their personal socio-economic status.

2. Addition to the Discussion Section (Limitations)

Finally, the SDI is based on national-level aggregates, which may obscure substantial within-country inequalities. For instance, individuals in low-SDI countries may, in some contexts, have access to high-quality healthcare, while marginalized populations

in high-SDI countries may face significant barriers to hypertension diagnosis and management. Future research should prioritize the use of individual-level and longitudinal data to refine risk estimation and better characterize the multifactorial relationships among HSBP, socioeconomic status, and IHD outcomes (lines 449-456).

Reviewer #2:

The manuscript titled "Global Trends and Disparities in Ischemic Heart Disease Attributable to High Systolic Blood Pressure, 1990–2021: Insights from the Global Burden of Disease Study" presents a comprehensive analysis of the global burden of ischemic heart disease (IHD) linked to high systolic blood pressure (HSBP). By leveraging the extensive Global Burden of Disease (GBD) 2021 dataset, the study aims to highlight trends, regional disparities, and demographic variations in IHD burden over three decades. While the manuscript is well-structured and provides valuable epidemiological insights, several critical issues need to be addressed to strengthen the robustness and interpretability of the findings.

General Comments:

Strengths:

The study utilizes a robust dataset (GBD 2021), ensuring a large sample size and global applicability.

The analysis of temporal trends and disparities across different sociodemographic index (SDI) categories adds value to the study.

The use of age-standardized rates (ASRs) and estimated annual percentage change (EAPC) enhances the comparability of results.

Weaknesses:

The manuscript lacks a clear justification for the focus on HSBP-related IHD, as opposed to other major modifiable risk factors for IHD (e.g., diabetes, smoking, obesity).

Re: We appreciate your thoughtful feedback and the opportunity to clarify our rationale for focusing on high systolic blood pressure (HSBP) as a major modifiable risk factor for ischemic heart disease (IHD). Below, we provide a clear justification based on existing literature and global disease burden trends.

Previous studies have assessed the global burden of IHD from multiple factors such as smoking, high LDL-C and high FPG [1,2]. However, these studies mainly focused on overall global trends and lacked detailed stratification of specific populations, such as different age groups, sexes, and geographic regions. Among all modifiable risk factors for IHD, HSBP has been consistently identified as the most significant contributor to the global burden of cardiovascular diseases, including IHD. GBD 2021 and previous studies confirm that HSBP accounts for a larger proportion of IHD-related deaths and disability-adjusted life years (DALYs) compared to other risk factors like diabetes, smoking, or obesity. Thus, given its high prevalence and direct pathophysiological impact, HSBP is a critical target for reducing IHD burden worldwide.

Numerous epidemiological studies have adopted GBD methodologies to examine single-risk factor contributions to IHD, and these studies have been widely recognized in leading journals [3-5]. Studies have individually analyzed the impact of high LDL-C, smoking, and high FPG on IHD burden, employing similar statistical modeling approaches[3-5]. However, despite the known high burden of HSBP, there remains a lack of in-depth studies specifically assessing HSBP-attributable IHD trends and disparities across different regions and populations. Additionally, attributable burden studies of HSBP on cardiovascular diseases, such as atrial fibrillation and stroke, have

been extensively conducted, further validating our analytical framework [6,7]. Our study aims to fill this gap by providing a focused analysis of HSBP-related IHD burden, allowing for more effective and targeted interventions. By addressing this underexplored yet highly impactful risk factor, our study makes a unique and valuable contribution to cardiovascular epidemiology.

We hope this clarification addresses your concern and strengthens the justification for our study. We appreciate your constructive feedback and look forward to your further comments.

References

[1] Safiri S, Karamzad N, Singh K, et al. Burden of ischemic heart disease and its attributable risk factors in 204 countries and territories, 1990-2019. Eur J Prev Cardiol. 2022;29(2):420-431. doi:10.1093/eurjpc/zwab213

[2] Wang Y, Li Q, Bi L, Wang B, Lv T, Zhang P. Global trends in the burden of ischemic heart disease based on the global burden of disease study 2021: the role of metabolic risk factors. *BMC Public Health*. 2025;25(1):310. Published 2025 Jan 24. doi:10.1186/s12889-025-21588-9

[3] Zhang L, Tong Z, Han R, et al. Global, Regional, and National Burdens of Ischemic Heart Disease Attributable to Smoking From 1990 to 2019. J Am Heart Assoc. 2023;12(3):e028193. doi:10.1161/JAHA.122.028193

[4] Du H, Shi Q, Song P, et al. Global Burden Attributable to High Low-Density Lipoprotein-Cholesterol From 1990 to 2019. Front Cardiovasc Med. 2022;9:903126. Published 2022 Jun 9. doi:10.3389/fcvm.2022.903126

[5] Shen N, Liu J, Wang Y, et al. The global burden of ischemic heart disease attributed to high fasting plasma glucose: Data from 1990 to 2019. Heliyon. 2024;10(5):e27065. Published 2024 Mar 6. doi:10.1016/j.heliyon.2024.e27065

[6] Jin Y, Wang K, Xiao B, et al. Global burden of atrial fibrillation/flutter due to high systolic blood pressure from 1990 to 2019: estimates from the global burden of disease study 2019. *J Clin Hypertens (Greenwich).* 2022;24(11):1461-1472. doi:10.1111/jch.14584

[7] Li J, Zhong Q, Yuan S, Zhu F. Global burden of stroke attributable to high systolic blood pressure in 204 countries and territories, 1990-2019. Front Cardiovasc Med. 2024;11:1339910. Published 2024 Apr 26. doi:10.3389/fcvm.2024.1339910

There is an overreliance on descriptive analysis without a deeper exploration of underlying causal mechanisms driving observed trends.

Re: Thank you for your thoughtful comment. In response, we would like to clarify that the **Introduction section (lines 66-85)** already outlines the key biological mechanisms through which HSBP contributes to IHD, including its interplay with other major cardiovascular risk factors. These pathophysiological insights offer essential context for interpreting the descriptive patterns reported in our study. Furthermore, we have explicitly acknowledged in the **limitations section (lines 443-449)** that residual confounding from unmeasured factors—such as smoking, diabetes, and obesity—may influence the estimated burden of HSBP-related IHD and were not fully adjusted for in our analysis.

The discussion on public health implications is underdeveloped, particularly regarding potential interventions and strategies tailored to different SDI groups.

Re: We appreciate your thoughtful feedback. In line with your suggestion, we have enriched the discussion by elaborating on the public health significance of our findings. The revised section outlines context-specific intervention strategies across SDI strata, taking into account variations in healthcare capacity, resource availability, and population needs. These strategies include enhancing hypertension awareness through community-based programs, expanding access to affordable antihypertensive therapies, leveraging non-physician providers, and incorporating digital health innovations (lines 381-424).

The manuscript does not sufficiently address limitations related to data quality and potential biases in the GBD dataset.

Re: Thank you for your observation. We have addressed this concern in the limitations section by acknowledging the potential biases associated with the GBD dataset. Specifically, we discuss the variability in data quality across regions, inconsistencies in blood pressure measurement protocols, and the challenges posed by underreporting of IHD—particularly in resource-limited settings. We also note that, despite the use of advanced statistical modeling techniques to harmonize data, these limitations may influence the accuracy of burden estimates (lines 434-443).

Detailed Comments:

Introduction:

The introduction effectively outlines the significance of IHD and its burden worldwide but would benefit from a more explicit rationale for focusing on HSBP as the primary risk factor. Including a comparative discussion of other key modifiable risk factors would improve contextual clarity.

Re: We appreciate your thoughtful suggestion. In the **revised Introduction (lines 66-85),** we have further clarified our rationale for selecting HSBP as the focal risk factor by emphasizing its direct pathophysiological link to IHD and its high global prevalence. While other modifiable risk factors such as smoking, diabetes, and dyslipidemia are also important contributors, HSBP stands out as a central upstream determinant. To address potential confounding, we have also included a statement in the **limitations section** (**lines 443-44)** acknowledging that the observed associations may be influenced by coexisting risk factors not explicitly adjusted for in our analysis.

The literature review should include more recent studies on the interplay between hypertension control policies and IHD burden across different global regions.

Re: Thank you for this valuable suggestion. In the revised manuscript, we have expanded the discussion to incorporate recent literature examining the impact of hypertension control policies on IHD burden across various global regions. Specifically, we highlight how differences in healthcare infrastructure, treatment accessibility, public awareness, and implementation of population-level interventions—such as salt-

reduction initiatives, community-based screening, and subsidized antihypertensive medications—have contributed to divergent IHD trends across SDI regions. This revision strengthens the contextual framing of our findings and emphasizes the importance of region-specific policy strategies for effective hypertension management (lines 381-424).

Methods:

The use of the GBD dataset is appropriate, but additional details on the methodology for calculating DALYs and ASRs should be provided. Specifically, how were missing or inconsistent data handled across the different countries included in the analysis?

Re: Thank you for your insightful comments. We provide more details in the Data Sources section about the methodology for calculating DALYs and for dealing with missing or inconsistent data for the different countries in the analysis. We also provide formulas for the calculation of ASRs in the Definitions section (lines 108-132).

The choice of statistical models, including smoothed curve modeling and Spearman correlation, should be better justified. Why were these methods selected over alternative approaches (e.g., multivariable regression models)?

Re: Thank you for your valuable feedback. We appreciate the opportunity to clarify our rationale for selecting Spearman's correlation and smoothed curve modeling over alternative approaches such as multivariable regression models.

Our study focuses on analyzing the relationship between a single independent variable (SDI) and a single dependent variable (ASRs of IHD attributable to HSBP). Given this,

we adopted the following approaches:

1. Spearman's correlation coefficient, a nonparametric method that is robust to deviations from normality and does not assume a linear relationship between variables. Spearman's correlation coefficient was chosen as it provides a robust, nonparametric measure of monotonic association between SDI and ASRs. Unlike Pearson's correlation, Spearman's correlation does not assume linearity and is well-suited for assessing rank-based associations in epidemiological data.

2. Smoothed curve modeling, which is well-suited for capturing potential nonlinear relationships between SDI and ASRs without assuming a predefined functional form. Smoothed curve modeling was used as a complementary method, allowing us to visualize localized trends and potential stage-specific variations that a single correlation coefficient might not fully capture. While Spearman's correlation quantifies the overall monotonic relationship, smoothed curve modeling helps to identify non-monotonic trends and possible inflection points along the SDI gradient.

Since our analysis focuses on assessing the correlation between only two variables, applying a multivariable regression model (which is designed for multiple independent variables) would not be appropriate in this context. Similarly, generalized additive models (GAMs), while useful for handling complex interactions, are not necessary for a straightforward bivariate analysis such as ours.

Moreover, we referenced several GBD study papers that used the same statistical methods [1-4].

References

[1] Sun P, Yu C, Yin L, et al. Global, regional, and national burden of female cancers in women of child-bearing age, 1990-2021: analysis of data from the global burden of disease study 2021. EClinicalMedicine. 2024;74:102713. doi:10.1016/j.eclinm.2024.102713

[2] Deng Y, Zhao P, Zhou L, et al. Epidemiological trends of tracheal, bronchus, and lung cancer at the global, regional, and national levels: a population-based study. J Hematol Oncol. 2020;13(1):98. doi:10.1186/s13045-020-00915-0

[3] Guo LH, Lin LZ, Zhou Y, et al. Global, regional, and national burden of ischemic heart disease attributable to ambient $PM_{2.5}$ from 1990 to 2019: An analysis for the global burden of disease study 2019. *Environ Res*. 2024;241:117635. doi:10.1016/j.envres.2023.117635

[4] Han S, Zhao S, Zhong R, et al. Global burden, trends, and disparities in kidney cancer attributable to smoking from 1990 to 2021. Front Public Health. 2025;12:1506542. doi:10.3389/fpubh.2024.1506542

The manuscript lacks sensitivity analyses to assess the robustness of the findings. Including sensitivity analyses that test the impact of different assumptions on the trends observed would enhance the credibility of the results.

Re: Thank you for your thoughtful feedback. We acknowledge the importance of sensitivity analyses in ensuring the robustness of our findings.

In our study, we primarily rely on GBD 2021 estimates, which incorporate extensive uncertainty quantification through 95% uncertainty intervals derived from multiple

modeling approaches and data sources. This built-in methodology ensures the reliability of the estimates and accounts for variations in input data and modeling assumptions. Additionally, as noted in our response regarding statistical model selection, we have applied appropriate statistical methods to assess associations, including Spearman's correlation and smoothed curve modeling. To further reinforce the robustness of our findings, we have included 95% confidence intervals in our smoothed curve models, which are visually represented in the results figures (shaded areas).

Furthermore, we have reviewed similar GBD-based studies, and they consistently apply the same statistical methods without conducting additional sensitivity analyses. We did not find a well-executed sensitivity analysis methodology. Given that our study follows an established methodological framework, additional sensitivity analyses are unlikely to provide further meaningful insights.

Based on these considerations, we believe our current approach sufficiently ensures the robustness of our findings. We appreciate your suggestion and will clarify these points in the manuscript to enhance transparency.

Results:

The presentation of findings is clear, but additional subgroup analyses (e.g., stratified by region, gender, and age group) would improve interpretability. For instance, were any regions or demographic groups found to have significantly different trends in HSBP-related IHD burden compared to the global average?

Re: Thank you for your valuable comment. In response, we expanded our subgroup

analyses to include age-specific trends across regions and addressed these findings in the **revised Discussion section (lines 274–288)**. Specifically, we interpret the observed rise in HSBP-related IHD burden among younger adults (aged 25–44) in regions such as East Asia, South Asia, and Sub-Saharan Africa, as well as the increased burden among individuals aged ≥75 years in selected areas. These trends are discussed in relation to demographic shifts, lifestyle changes, and health system factors, underscoring their implications for age-tailored prevention and control strategies (lines 368-374).

The discussion on increasing ASRs in low-SDI regions needs further elaboration. What specific socioeconomic or healthcare system factors may be driving these increases?

Re: Thank you for this important observation. In the revised Discussion section (lines 392-401), we have expanded our explanation of the socioeconomic and healthcare system factors contributing to rising ASRs in low-SDI regions. Specifically, we discuss limited healthcare infrastructure, low hypertension awareness, inadequate screening programs, financial barriers to antihypertensive treatment, and weak primary care systems as key contributors. We also highlight the impact of rapid urbanization, dietary shifts toward high-sodium and processed foods, and lack of public health initiatives targeting early blood pressure control. These factors together likely account for the increasing burden of HSBP-related IHD in low-resource settings.

The correlation analyses presented in the results should include effect size

interpretations to aid in understanding the magnitude of associations.

Re: Thank you for this thoughtful suggestion. In response, we have added a brief explanation of effect size interpretation in the statistical **methods section (lines 186-188),** clarifying how correlation coefficients (r) are categorized (e.g., weak, moderate, strong, very strong). Additionally, we have updated the Results section to indicate that the observed negative correlations between SDI and both age-standardized DALYs (r = - 0.308) and ASDR (r = - 0.262) represent weak inverse associations. This clarification helps contextualize the magnitude of the associations and enhances the interpretability of our findings.

Discussion:

The discussion should incorporate a more in-depth analysis of policy implications. Given the findings, what specific interventions should be prioritized in high-burden regions?

Re: Thank you for your valuable suggestion. In the **revised Discussion section (lines381-424)**, we have expanded our analysis of policy implications by incorporating region-specific recommendations based on SDI levels and observed burden trends. For high-burden, low- and middle-SDI regions, we emphasize the need to strengthen primary care infrastructure, improve access to cost-effective antihypertensive medications through national subsidy or essential medicine programs, and implement community-based screening initiatives. Additionally, task-shifting models involving non-physician health workers and mobile health (mHealth) strategies are highlighted as feasible, scalable solutions. For high-SDI regions, we recommend enhancing long-term

treatment adherence through digital health interventions and reinforcing integrated cardiovascular risk management approaches. These tailored strategies are grounded in our findings and aim to inform practical, evidence-based policy responses.

More emphasis should be placed on explaining the disparities observed in different SDI regions. The manuscript should explore whether differences in healthcare access, medication adherence, or public health policies contribute to the findings.

Re: Thank you for your insightful comments. In response, we have expanded the **Discussion section (lines383-424)** to more clearly address the underlying drivers of regional disparities in HSBP-related IHD burden across SDI levels. Specifically, we discuss how variations in healthcare infrastructure, access to antihypertensive medications, affordability, treatment adherence, and implementation of public health policies contribute to the divergent trends observed across regions. In high-SDI regions, widespread screening, subsidized medications, and preventive health programs have contributed to improved hypertension control. Conversely, low- and middle-SDI regions face persistent challenges such as limited primary care capacity, high out-of-pocket costs, and low hypertension awareness, which may explain the rising ASRs observed in these settings. These contextual factors are now more thoroughly integrated into our interpretation of the results.

The limitations section should explicitly discuss potential biases in the GBD dataset, such as underreporting of IHD in certain regions or inconsistencies in blood pressure

measurement methodologies across countries.

Re: Thank you for your valuable comment. In response, we have revised the Limitations section to explicitly address potential biases related to the GBD dataset. Specifically, we now acknowledge the possibility of underreporting of IHD in resource-limited regions, as well as inconsistencies in blood pressure measurement methodologies across countries. These issues may introduce systematic bias into the burden estimates, and this clarification enhances the transparency and robustness of our findings **(see revised lines 434-443).**

Conclusion:

The conclusion is concise but should better emphasize actionable recommendations for policymakers and healthcare providers. Consider highlighting the importance of future research to examine interventions aimed at reducing HSBP and mitigating its impact on IHD.

Re: Thank you for your insightful comments. We have modified the conclusions.

Modified conclusions:

"This study highlights the growing global burden of HSBP-related IHD, with significant disparities across age groups, genders, and regions. While age-standardized rates have declined, the absolute burden remains high, particularly in low- and middle-SDI regions, where limited healthcare access and low treatment adherence drive rising trends. To address this, high-SDI regions should focus on improving long-term hypertension control through digital health tools and adherence strategies, while low-

and middle-SDI regions must prioritize expanding access to affordable antihypertensive medications and integrating hypertension management into primary care. Future research should assess the effectiveness of targeted interventions and explore policy-driven strategies to enhance hypertension prevention and treatment, ultimately reducing IHD burden worldwide.”

Reviewer #3:

The study comprehensively described global data on the risk of ischemic heart disease attributable to high systolic blood pressure, provided valuable data on regional variations. The results are scientifically sound and could impact health care. I would suggest the R code they used to get the study results with outputs. It would help future researchers to replicate the results of the study. Also, I think it will help in dissemination of the paper among the scientific society.

Re: Thank you for your insightful comments. We provide the R code used for the statistical analysis in the Supplementary Materials section.
