# Supplementary material for: Global trends and disparities in ischemic heart disease attributable to high systolic blood pressure, 1990–2021: Insights from the global burden of disease study
Source: PLoS One. 2025 Jun 12;20(6):e0324073. doi: 10.1371/journal.pone.0324073 (PMC12161563; doi:10.1371/journal.pone.0324073)
Supplement: Attachment — Submitted filename: Response to Reviewers_2.pdf [file pone.0324073.s006.pdf]

Response to Reviewers

Journal Requirements:

Please review your reference list to ensure that it is complete and correct. If you have cited papers that have been retracted, please include the rationale for doing so in the manuscript text, or remove these references and replace them with relevant current references. Any changes to the reference list should be mentioned in the rebuttal letter that accompanies your revised manuscript. If you need to cite a retracted article, indicate the article's retracted status in the References list and also include a citation and full reference for the retraction notice.

Re: Thank you for your editorial guidance regarding the reference list. We have thoroughly reviewed all cited literature. All references have been cross-checked for accuracy and current status.

In addition, as per a reviewer's suggestion, we have cited the recent article by Cesaro et al. (2025) [DOI: 10.1007/s40292-025-00705-0] to highlight the challenges in cardiovascular risk perception and decision-making related to hypertension in primary prevention. This citation has been incorporated into the revised manuscript and added to the updated reference list. See reference 20.

Reviewers' comments:

Reviewer #2:

Justification for Focus on HSBP: The authors have expanded the rationale for selecting HSBP, though the introduction would benefit from a stronger link between underestimated HSBP impact and real-world cardiovascular risk perception among clinicians.

Suggested addition: Cite the paper by Cesaro et al., titled "Discrepancies Between Physician-Perceived and Calculated Cardiovascular Risk in Primary Prevention: Implications for LDL-C Target Achievement and Appropriate Lipid-Lowering Therapy" ([doi:10.1007/s40292-025-00705-0]) to highlight the persistent under-recognition of hypertension in cardiovascular risk assessment and decision-making.

Re: We sincerely appreciate your valuable comments. To strengthen the rationale for highlighting HSBP, we have added a sentence at the end of the fifth paragraph in the introduction, referencing Cesaro et al. (2025), to emphasize that elevated blood pressure is frequently under-recognized in clinical cardiovascular risk assessment.

Revised introduction: *Although HSBP is a well-established contributor to ischemic heart disease, it remains a "silent burden" in clinical practice—frequently under-recognized in cardiovascular risk stratification and undertreated in primary prevention. Recent findings by Cesaro et al. highlight a persistent disconnect between physician-estimated and calculated cardiovascular risk, indicating that elevated blood pressure is often undervalued in clinical decision-making, potentially delaying timely*

intervention. (Lines 94-99).

Adding a brief note acknowledging that causal inference is limited by the ecological nature of GBD data would improve transparency.

Re: Thank you for your thoughtful comment. We have added a sentence in the limitations section to clarify that causal inference is constrained by the ecological nature of the data.

Revised limitations section: " *Third, the cross-sectional design of this study limits causal inference. It is not possible to definitively establish whether elevated SBP preceded the development of IHD or whether observed associations were confounded by preexisting comorbid conditions. Moreover, the ecological nature of GBD estimates—derived from population-level rather than individual-level data—further limits the ability to draw causal inferences across countries or subgroups. (Lines 448-453)*"

Public Health Recommendations: Reviewer #2 requested expanded intervention strategies. The revised manuscript now offers policy-relevant proposals stratified by SDI level, which is a welcome improvement.

Re: We appreciate the reviewer's recognition of this revision.

Sensitivity Analysis: The authors provided a reasonable justification for not conducting formal sensitivity analyses, citing the use of GBD uncertainty intervals.

This should be briefly mentioned in the limitations section for clarity.

Re: We appreciate your suggestion. To enhance clarity, we have added a brief note in the limitations section acknowledging that while no formal sensitivity analyses were performed, the use of 95% uncertainty intervals within the GBD framework provides an indication of estimate variability.

Revised limitations section: "Additionally, while no formal sensitivity analyses were performed, the 95% uncertainty intervals generated by the GBD modeling framework offer an indication of variability around the estimates." (Lines 463-466).

Detailed Comments:

Introduction:

Integrate the above-cited reference to reinforce the concept that hypertension is often under-recognized or undervalued relative to its true impact on IHD. Reframe the discussion of HSBP as a "silent burden" that may be systematically underestimated in risk stratification tools.

Re: Thank you for your thoughtful comment. To better contextualize the clinical relevance of HSBP, we have added a new sentence in the Introduction highlighting its under-recognition as a "silent burden" and citing the study by Cesaro et al. (2025) to reflect the discrepancy between perceived and calculated cardiovascular risk in primary prevention.

Revised introduction: "*Although HSBP is a well-established contributor to ischemic*

heart disease, it remains a "silent burden" in clinical practice—frequently under-recognized in cardiovascular risk stratification and undertreated in primary prevention. Recent findings by Cesaro et al. highlight a persistent disconnect between physician-estimated and calculated cardiovascular risk, indicating that elevated blood pressure is often undervalued in clinical decision-making, potentially delaying timely intervention." (Lines 94-99).

Methods:

The description of SDI assignment at the country level is now clear. Statistical rationale for Spearman and smoothed curve modeling is well-articulated.

Re: We appreciate your positive feedback.

Results:

The age-stratified and region-specific subgroup trends are now clearly presented and contextualized.

Re: We acknowledge your favorable assessment.

Consider annotating graphs to highlight key divergence points among SDI categories.

Re: Thank you for your helpful suggestion. To improve interpretability, we have added visual annotations to Figure 6 to indicate key divergence points in the association between SDI and age-standardized IHD burden.

Revised figure legend (Figure 6):

“Fig 6 SDI-based age-standardized rates of DALYs and ASDR for IHD attributable to HSBP. (A) SDI-based age-standardized rates of DALYs in 21 regions from1990 to 2021. (B) SDI-based ASDR in 21 regions from1990 to 2021. (C) SDI-based age-standardized DALYs across countries in 2021. (D) SDI-based ASDR across countries in 2021. Arrows point to major inflection points (SDI ≈ 0.7) where IHD burden begins to decline with increasing SDI. SDI: Socio-demographic Index; DALYs: Disability-adjusted life years; ASR: Age-standardized rate.”

Discussion:

The extended analysis of sex- and region-specific disparities is stronger. Emphasize how findings support global and local initiatives for hypertension control.

Re: We appreciate your thoughtful suggestion. In response, we have expanded the Clinical Significance section to highlight the practical implications of our findings for global and region-specific hypertension control efforts.

Revised Limitations and Clinical Significance: “*Despite these limitations, our analysis provides meaningful insights into the demographic and geographic variability of IHD burden attributable to hypertension. By identifying high-risk populations and regions, the findings offer a basis for targeted interventions and policy development. The observed parabolic relationship between SDI and disease burden suggests that uniform strategies may be suboptimal. In lower-SDI settings, efforts should focus on early detection, treatment accessibility, and primary care integration. In higher-SDI contexts, optimizing adherence, promoting healthy lifestyles, and addressing care gaps*

remain key. Aligning hypertension control strategies with health system capacity and socioeconomic conditions may help reduce global disparities in cardiovascular outcomes." (Lines 468-477).

Limitations

Acknowledge the inability to infer causality from ecological-level GBD data.

Re: Thank you for this valuable suggestion. As suggested, we have clarified in the Limitations section that the ecological design of the GBD data.

"*Moreover, the ecological nature of GBD estimates—derived from population-level rather than individual-level data—further limits the ability to draw causal inferences across countries or subpopulations."* (lines 452-454).

Note that despite robust modeling, within-country inequalities in SDI and BP control are not captured

Re: Thank you for this insightful comment. We have revised the Limitations section to emphasize that national-level indicators such as SDI and blood pressure may obscure important within-country disparities.

"Finally, the use of national-level aggregates—such as SDI and blood pressure data—may obscure substantial within-country disparities, particularly in large or socioeconomically diverse countries. For example, individuals in low-SDI countries may, in some contexts, have access to high-quality healthcare, while marginalized groups in high-SDI countries may face significant barriers to hypertension diagnosis

and treatment. Future research should incorporate individual-level and longitudinal data to improve risk estimation and better elucidate the complex interactions among HSBP, socioeconomic status, and IHD outcomes." (Lines457-464).

Conclusion:

Now appropriately focused and policy-oriented.

Re: Thank you for the positive feedback.
